# Unusual Localization of Clostridium Difficile Infection in an Isolated Segment of the Descending Colon in a Critical Care Patient

**DOI:** 10.1155/2012/482930

**Published:** 2012-12-18

**Authors:** Evgeni Brotfain, Leonid Koyfman, Amit Frenkel, Jochanan G. Peiser, Abraham Borer, Benjamin F. Gruenbaum, Alexander Zlotnik, Moti Klein

**Affiliations:** Department of Anesthesiology and Critical Care, Soroka Medical Center, Ben-Gurion University of the Negev, 84105 Beer Sheva, Israel

## Abstract

Unrecognized severe pseudomembranous colitis may become life threatening. A typical Clostridium difficile infection is associated with involvement of the colon; however, small bowel disease has also been described. Here, we present a case of a 48-year-old man with Clostridium difficile colitis of an isolated segment in the descending colon treated by a novel catheter intraluminal antibiotic irrigation. The intraluminal antibiotic irrigation was performed through a Foley catheter inserted into the isolated mucus fistula. The patient recovered after three weeks of intraluminal vancomycin (250 mg diluted in 150 ml of normal saline  x Q6) and metronidazole (500 mg  x Q8). Both antibiotics were given into the mucus fistula over 30 min. The patient was discharged from the unit four weeks after admission. This novel technique, in which the antibiotic was administered through an inserted intraluminal Foley urinary catheter, may be an efficient and safe alternative when conventional routes cannot be implemented.

## 1. Introduction

*Clostridium difficile* is a gram-positive, toxin produced, anaerobic microorganism [1]. A simple asymptomatic carrier could rapidly progress to subsequent pseudomembranous colitis and even fulminant toxic megacolon, which is associated with a high mortality rate [2]. Severely ill patients could be detected by an elevated white cell count and fever. This may be followed by an increase in serum creatinine level and hemodynamic instability [2, 3]. A typical Clostridium difficile infection is associated with involvement of the colon [2]; however, small bowel disease has also been described [1, 2]. Initial measures include stopping antibiotic treatment, supportive care, and peroral metronidazole management [3, 4]. Tremendous clinical deterioration and life-threatening gastrointestinal complications might need intensive care management and even emergency surgery. Here, we present a case of Clostridium difficile colitis of an unusual intestinal localization treated by a novel catheter intraluminal antibiotic irrigation.

## 2. Case Report

A 48-year-old male was admitted to the internal medicine ward with chronic pancytopenia secondary to megaloblastic anemia. He had a past medical history of hypertension, dyslipidemia, and a history of smoking. 

Three days after admission, septic bursitis of the right olecranon was diagnosed and the patient was treated by surgical drainage of the abscess and a week-long course of antibiotic therapy (fluoroquinolone and penicillin groups). During the next 72 hours, a high fever and abdominal distention were noted. Free air in the abdomen was shown on urgently performed CT and the patient was transferred to the operating room. 

Exploration of the abdomen revealed an ischemic area in the cecum with a perforation. Right hemicolectomy with ileostomy was performed. The remaining large intestine was brought out to the abdominal wall as a mucous fistula at the left upper quadrant. 

During the next four days in the intensive care unit, the patient continued to be septic with highly elevated temperatures (39.2°C) and extensive leukocytosis (37000 cells/uL). During this time, intensive supportive management included the administration of vasopressors and broad spectrum antibiotics.

**Figure 1:**
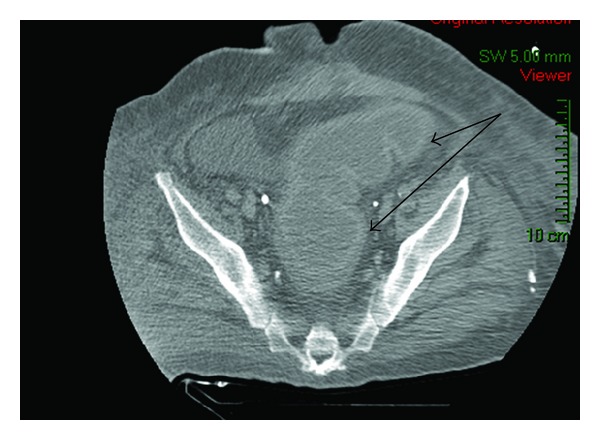
CT abdomen of a patient with Clostridium difficile colitis. The image shows dilated descending loop with thickening of wall (see black arrows).

On a detailed workup of sepsis, stool sample cultures for Clostridium difficile toxins from the rectum and end ileostomy were performed. Diagnosis of colitis was made by a positive Clostridium difficile toxin in the rectal sample and was confirmed by CT findings of a thickened, dilated, and distended colonic wall (mucus fistula segment) (Figure 1). Clostridium difficile culture from the ileostomy was negative. Moreover, a pathological examination disclosed cecal perforation, acute peritonitis, and a tubulovillous adenoma in the cecum which was located 3 centimeters distal to the point of perforation. No evidence of colitis was encountered in the removed segment of the right colon.

Infection disease was consulted on how to treat the colonic descending loop. A novel intraluminal antibiotic irrigation through a Foley catheter inserted into the isolated mucus fistula was implemented. The patient recovered after three weeks of intraluminal vancomycin (250 mg diluted in 150 mL of normal saline x Q6) and metronidazole (500 mg x Q8). Both antibiotics were given into the mucus fistula over 30 min. The patient was discharged from the unit four weeks after admission. 

## 3. Discussion

Primary diagnosis of Clostridium difficile colitis is based on the presentation of diarrhea with positive toxin stool cultures. Endoscopic examination may also detect typical macroscopic pseudomembrane findings of the colonic wall. CT findings can be helpful in confirming the diagnosis, showing thickening, dilation, and potential perforation of the colon [2, 5–7].

This patient presented with classic clinical features of severe Clostridium difficile, marked by a high-grade fever and extensive leukocytosis. However, the perforated caecum had no signs of mucus edema, congestion, or pseudomembrane formation on biopsy and the end-ileostomy Clostridium difficile toxin test was negative. 

There was a high level of suspicion of end-ileostomy infection, given a previous history of antibiotic use, which led us to the diagnosis of Clostridium difficile colitis in the isolated descending left colon. Our suspicions were confirmed by a positive Clostridium difficile toxin test from the isolated colon and remarkable CT findings.

This unusual localization of Clostridium difficile infection has been previously described [8–10]. Causey et al. reported three cases of Clostridium difficile enteritis developed after total colectomy [11]. Moreover, it has been recognized in surgical literature as secondary pouchitis of the ileal pouch-anal anastomosis after colorectal surgery. However, in our patient, Clostridium difficile colitis developed only in the isolated colon loop. 

Al-Mufarrej et al. described a curious case of a 47-year-old male who developed Clostridium difficile infection in an interposed reconstructed colon after esophagectomy [8]. In addition, Oppermann et al. reported about isolated Clostridium difficile colitis in the ascending colon after transverse colon loop colostomy [12]. The overall collection of cases strongly supports the ability of previously colonized heat-resistant Clostridium difficile spores to convert to vegetative forms, capable of rapidly progressing to severe systemic disease even in isolated segments of the colon.

The challenge of an appropriate route of therapy was elusive. We started to treat our patient in accordance with the well-described guidelines for severe Clostridium difficile. However, oral metronidazole and vancomycin together with metronidazole intravenous therapy were not suitable in our case considering the isolated location. We decided to implement a novel method of administration through a Foley urinary catheter inserted into a mucus fistula end. The rapid improvement of our patient demonstrated that this route of administration could be considered in future cases with a similar presentation. 

## 4. Conclusion

Unrecognized severe pseudomembranous colitis may become life threatening. Unusual localizations must be considered in patients after intestinal resection surgeries. The workup of sepsis in critical illness population should include stool samples from different intestinal sites and also isolated segments of intestine. In the case described, local intraluminal antibiotic therapy was defiant. We present a novel technique, in which the antibiotic was administered through an inserted intraluminal Foley urinary catheter as an efficient and safe alternative when conventional routes cannot be implemented.
